# The Prognostic Value of Preoperative Total Cholesterol in Surgically Treated Oral Cavity Cancer

**DOI:** 10.3390/biomedicines12122898

**Published:** 2024-12-19

**Authors:** Yao-Te Tsai, Ming-Hsien Tsai, Adarsh Kudva, Andrea De Vito, Chia-Hsuan Lai, Chun-Ta Liao, Chung-Jan Kang, Yuan-Hsiung Tsai, Cheng-Ming Hsu, Ethan I. Huang, Geng-He Chang, Ming-Shao Tsai, Ku-Hao Fang

**Affiliations:** 1 Department of Otorhinolaryngology-Head and Neck Surgery, Chang Gung Memorial Hospital, Chiayi 613016, Taiwan; yaote1215@gmail.com (Y.-T.T.); scm00031@gmail.com (C.-M.H.); ehuang.mdphd@gmail.com (E.I.H.); genghechang@gmail.com (G.-H.C.); b87401061@gmail.com (M.-S.T.); 2 College of Medicine, Chang Gung University, Taoyuan 330036, Taiwan; b9302094@cgmh.org.tw (M.-H.T.); chiahsuan7092@gmail.com (C.-H.L.); liaoct@cgmh.org.tw (C.-T.L.); handneck@gmail.com (C.-J.K.); russell.tsai@gmail.com (Y.-H.T.); 3 Department of Otorhinolaryngology-Head and Neck Surgery, Chang Gung Memorial Hospital, Kaohsiung 833253, Taiwan; 4 Department of Oral and Maxillofacial Surgery, Manipal College of Dental Sciences, Manipal Academy of Higher Education, Manipal 576104, India; adarsh.kudva@manipal.edu; 5 Ear Nose Throat (ENT) Unit, Department of Surgery, Forli Hospital Health Local Agency of Romagna, 47121 Forli, Italy; drandreadevito@gmail.com; 6 Department of Radiation Oncology, Chang Gung Memorial Hospital, Chiayi 613016, Taiwan; 7 Department of Otorhinolaryngology-Head and Neck Surgery, Chang Gung Memorial Hospital, Taoyuan 333423, Taiwan; 8 Department of Diagnostic Radiology, Chang Gung Memorial Hospital, Chiayi 613016, Taiwan

**Keywords:** oral squamous cell carcinoma, total cholesterol, biomarkers, prognosis, nomogram

## Abstract

Background: With growing evidence linking lipid profile changes to tumor development and cancer prognosis, we investigated the prognostic significance of preoperative serum total cholesterol (TC) levels in patients with oral cavity squamous cell carcinoma (OSCC) undergoing surgical treatment. Methods: We conducted a retrospective observational study involving 310 patients with primary OSCC who received surgery at our hospital from January 2009 to December 2018. Receiver operating characteristic curve analysis was performed to determine the optimal preoperative TC cutoff value, with the Youden Index employed as the optimization criterion to maximize the sum of sensitivity and specificity. Variables with *p* < 0.1 in the univariable analysis were included in the multivariable Cox regression model, and stepwise selection was used to identify the optimal subset of prognostic factors for overall survival (OS) and disease-free survival (DFS). Results: An optimal TC cutoff of 157 mg/dL was established. Patients with TC < 157 mg/dL exhibited significantly lower 5-year rates of OS and DFS (*p* < 0.001 and *p* = 0.006, respectively). Multivariable analysis confirmed that TC < 157 mg/dL represented an independent prognostic factor for reduced OS and DFS rates. Subgroup analyses reinforced the consistent prognostic significance of TC. We also constructed a nomogram (concordance index: 0.74) to provide personalized OS predictions, enhancing the clinical utility of TC. Conclusions: Preoperative TC appears to be a significant prognostic factor for OS and DFS after OSCC surgery. Routine TC assessment facilitates the development of nomograms for personalized survival predictions, supports clinicians in tailoring treatment strategies, and guides nutritional or metabolic interventions to enhance patient outcomes. Further multicenter prospective studies are needed to validate our findings.

## 1. Introduction

In 2020, approximately 377,000 people worldwide received a diagnosis of oral cavity squamous cell carcinoma (OSCC), and around 177,000 succumbed to the disease [1]. Despite advancements in diagnostics and treatments, the prognosis for OSCC remains unsatisfactory, primarily due to delays in diagnosis, frequent locoregional recurrence, and a high incidence of second primary cancer [2]. Ablative surgery remains the mainstay of therapy for OSCC, with adjuvant therapy recommended when pathological analysis reveals high-risk factors [3]. Critical prognostic indicators for OSCC include lymphovascular invasion (LVI), perineural invasion (PNI), and poor differentiation (P–D) [4,5,6]. Nevertheless, as these pathological factors are typically assessed post-surgery, the inclusion of preoperative prognostic markers alongside conventional tumor–node–metastasis (TNM) staging could aid in early patient stratification, enabling personalized treatment planning and more effective selection of adjuvant therapies.

Serum cholesterol, an essential marker for lipid metabolism, plays a crucial role in maintaining cell membrane integrity and regulating functions such as signal transmission across cell membranes and cell adhesion to the surrounding matrix. Recent studies suggest that alterations in lipid metabolism are significant in OSCC development and progression [7], and serum total cholesterol (TC) has garnered attention for its potential role in tumorigenesis [8,9]. Research across various types of cancer, such as pancreatic, gastric, prostate, cervical, renal cell carcinoma, and non-small cell lung cancer, has examined TC as a prognostic marker [10,11,12,13,14,15]. Studies have shown that patients with OSCC generally exhibit lower mean serum TC levels compared to individuals without OSCC [16,17]. A retrospective study of 512 patients with OSCC found that high low-density lipoprotein (LDL) cholesterol, rather than TC, was an independent protective factor for disease-specific survival [18]. However, it is important to note that this study primarily focused on early-stage OSCC (T1/2N0M0), with tongue cancer accounting for the majority of cases (78.1%), potentially limiting its generalizability to broader OSCC populations. Conversely, a meta-analysis involving 24,655 patients with cancer suggested that serum TC levels—but not LDL cholesterol—were significantly associated with disease-free survival (DFS) and overall survival (OS), with higher TC levels demonstrating a protective effect [19]. However, only two studies in this meta-analysis focused on head and neck cancer. We believe these discrepancies may stem from variations in study populations, cancer stages, tumor subsites, and methodologies, which highlights the need for further studies to comprehensively assess the prognostic role of TC in OSCC. In this retrospective study, we explored the TC’s prognostic significance in patients who underwent surgical treatment for OSCC. Considering TC’s key role in the rapid proliferation of cancer cells, cell membrane synthesis, and energy production [7], we hypothesized that preoperative serum TC levels would significantly correlate with OSCC prognoses.

## 2. Materials and Methods

### 2.1. Study Cohort

We retrospectively analyzed clinical data from 331 patients who underwent ablative surgery for primary OSCC at Linkou Chang Gung Memorial Hospital between January 2009 and December 2018. Patients were included if they had a histopathologically confirmed diagnosis of OSCC and had undergone curative resection. Patients were excluded if they had prior neoadjuvant treatment (*n* = 3), had a history of cancer or severe liver disease (*n* = 5), had presented with synchronous cancer or unresectable OSCC at diagnosis (*n* = 5), or had taken medications known to affect lipid metabolism (*n* = 8). After applying these criteria, 310 treatment-naïve patients were enrolled. This study adhered to the Declaration of Helsinki and its amendments, and the protocol was approved by the Institutional Review Board of Chang Gung Memorial Hospital (IRB number: 202400093B0C601). Given the study’s retrospective nature, the requirement for informed consent was waived by the Institutional Review Board.

### 2.2. Study Variables

We collected detailed clinical data from the patients’ electronic medical records, including tumor subsite, sex, age, pathological cancer staging (determined based on the eighth edition of the American Joint Committee on Cancer Staging Manual), depth of invasion (DOI), closest resection margin, tumor grade, and status of LVI, PNI, and extranodal extension. Patients were categorized based on whether they had none, one, or two or more of the following habits: alcohol consumption, cigarette smoking, and betel nut chewing [20,21]. Laboratory data, such as serum TC and albumin levels, were collected within one week prior to surgery. TC was measured using the standard enzymatic colorimetric method from Roche Diagnostics (Rotkreuz, Switzerland), while serum biochemistry data were gathered using the automated Roche Cobas 8000 system from Hitachi (Rotkreuz, Switzerland). Moreover, we employed the Sysmex SE-9000 system (Kobe, Japan) to assess peripheral blood cell counts. Comorbidities were categorized using the Charlson Comorbidity Index (CCI).

### 2.3. Treatment and Follow-Up Plans

Prior to surgery, the patients underwent routine cancer staging workups, including MRI or CT of the head and neck, chest X-rays, abdominal sonography, and a nuclear bone scan, as per our institutional guidelines [3]. All patients underwent curative surgery, accompanied by neck dissection when indicated; resection defects were reconstructed by plastic surgeons. Adjuvant therapy was administered within six weeks post-surgery for patients who met the institutional criteria [3]. This included intensity-modulated radiotherapy (total dose: 60–66 Gy in 2-Gy fractions daily for 5 days per week) and chemotherapy (intravenous cisplatin at either 100 mg/m^2^ every three weeks or 40 mg/m^2^ weekly), as indicated.

The patients were followed up at 2–3-month intervals during the initial two years and at four–six-month intervals thereafter. Each follow-up included physical examinations, periodic imaging, and fiberoptic laryngoscopy. If clinical abnormalities were detected, additional MRI or CT scans were conducted along with a biopsy. In cases of locoregional recurrence, salvage resection or chemoradiotherapy was administered. The outcome measures comprised OS (derived as the interval spanning from the date of surgery to either the date of death by any cause or censoring at the last follow-up, whichever came first) and DFS (derived as the interval spanning from the date of surgery to the date of tumor recurrence, metastasis, censoring, or death).

### 2.4. Statistical Analysis

We used the Shapiro–Wilk test to appraise data normality. Here, we report median and interquartile range values for nonnormally distributed continuous variables, in addition to reporting counts and percentages for categorical variables. We determined the optimal TC cutoff value using receiver operating characteristic curve analysis and deriving the Youden index. The aforementioned clinical variables’ associations with survival outcomes were assessed using the log-rank test and the Kaplan–Meier method. Univariable analysis was conducted using the Cox proportional hazards model to identify potential prognostic factors, and variables with *p*-values < 0.1 were included in the multivariable analysis. Stepwise selection was used to identify the optimal subset of prognostic factors, and multicollinearity was assessed using the variance inflation factor. We deemed a two-sided *p*-value of < 0.05 statistically significant. The mentioned analyses were executed through SPSS (v23.0; IBM; Armonk, NY, USA) and R software (version 4.2.0).

We derived a nomogram to predict the 3-year and 5-year rates of OS, incorporating significant prognostic factors identified in the multivariable analysis, using the rms package (R version 5.1-0; Vanderbilt University, Nashville, TN, USA) [22]. The nomogram’s performance was compared to the TNM staging system using the concordance index, and calibration plots were used to evaluate the agreement between predicted OS and observed outcomes.

## 3. Results

### 3.1. Patient Characteristics

Table 1 provides an overview of patient characteristics. The median follow-up duration was 41 months (interquartile range: 21–67 months), during which there were 85 deaths. Most patients were male (n = 280, 90.3%), with tongue and buccal cancers being the most frequent tumor subsites. Half of the patients were diagnosed with stage IV OSCC (n = 156, 50.3%), and approximately one-third (n = 109, 35.2%) had neck lymph node metastasis. In terms of treatment, 149 patients (48.1%) underwent surgery alone, 44 (14.2%) received adjuvant radiotherapy, and 117 (37.7%) received adjuvant chemoradiotherapy. Approximately half of the patients (n = 167, 53.9%) had no significant comorbidities (CCI of 0), while the rest (n = 143, 46.1%) had at least one comorbidity according to the CCI classification.

### 3.2. Determining the Optimal Total Cholesterol Cutoff

**Figure 1:**
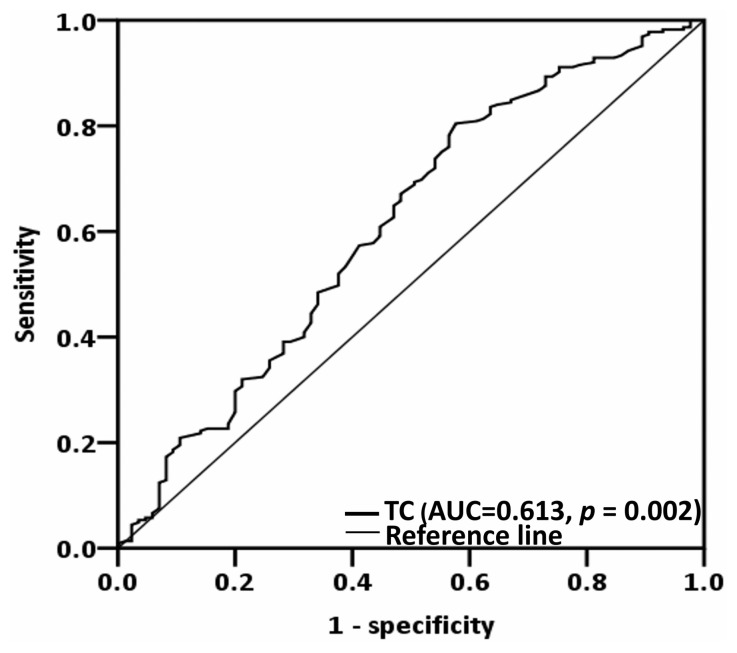
Optimal TC cutoff value, as derived via executing receiver operating characteristic curve analysis. Abbreviation: TC, total cholesterol.

The literature shows a range of TC cutoff values for patients with cancer [10,11,13,14,15]. To determine the optimal TC cutoff for managing OSCC, we conducted receiver operating characteristic curve analysis, using OS as the primary outcome. We identified 157 mg/dL as the optimal TC cutoff value (sensitivity = 80.4%; specificity = 43.7%; *p* = 0.002; Figure 1). Based on this cutoff value, the patients were divided into low-TC (<157 mg/dL) and high-TC (≥157 mg/dL) groups for further analysis. Then, we explored TC’s potential links with various clinicopathological variables in OSCC (Table 1). We noted that low TC was significantly associated with age ≥ 65 (*p* = 0.010), T3 or T4 tumors (*p* = 0.020), the presence of PNI (*p* = 0.049), and DOI ≥ 10 mm (*p* = 0.018).

### 3.3. Prognostic Factors for Overall Survival

**Figure 2:**
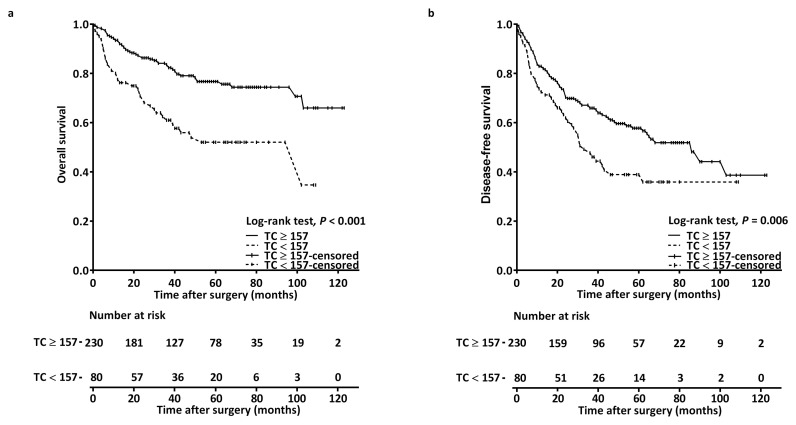
Kaplan–Meier curves for (**a**) overall survival and (**b**) disease-free survival, stratified by preoperative TC cutoff. Abbreviation: TC, total cholesterol.

The estimated 1-year, 3-year, and 5-year rates of OS were 80.1%, 62.5%, and 52.0%, respectively, in the low-TC group, and 93.4%, 84.1%, and 76.7%, respectively, in the high-TC group (log-rank *p* < 0.001; Figure 2a).

**Figure 3:**
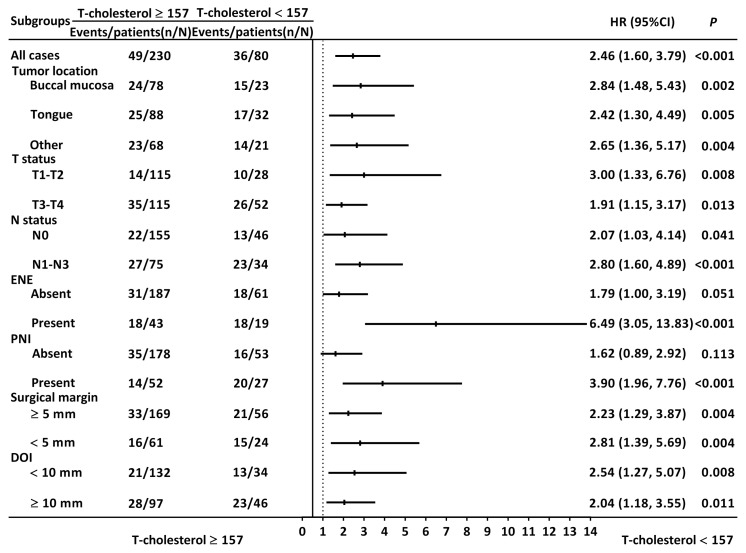
Subgroup analysis assessing the discriminatory capacity of TC for overall survival. Abbreviations: CI, confidence interval; DOI, depth of invasion; ENE, extranodal extension; HR, hazard ratio; PNI, perineural invasion; T-cholesterol, total cholesterol.

Table 2 shows the associations between OS and clinicopathological variables. Our univariable Cox analysis indicated that TC < 157 mg/dL was linked to poorer OS (HR = 2.463; 95% CI = 1.601–3.792; *p* < 0.001). Other factors significantly associated with OS included stage III–IV disease, the requirement for adjuvant therapy, PNI, LVI, P–D, and resection margin < 5 mm. To further substantiate the prognostic significance of TC in OSCC and mitigate other confounding factors’ effects, we conducted stepwise multivariable Cox regression analyses. Our findings confirmed TC as an independent prognostic factor for OS (HR = 2.192; 95% CI = 1.420–3.384; *p* < 0.001), along with stage III–IV disease, LVI, and P–D (Table 3). Subgroup analysis showed that TC’s prognostic value remained significant regardless of tumor subsite, T and N status, resection margin, or DOI (Figure 3).

### 3.4. Prognostic Factors for Disease-Free Survival

The estimated 1-year, 3-year, and 5-year rates of DFS were 73.8%, 49.1%, and 38.9%, respectively, in the low-TC group, and 82.9%, 67.1%, and 57.8%, respectively, in the high-TC group (log-rank *p* = 0.006; Figure 2b). Table 3 details the associations between DFS and clinicopathological variables. Our univariable Cox analysis revealed that TC < 157 mg/dL was significantly linked to poorer DFS (HR = 1.630; 95% CI = 1.144–2.324; *p* = 0.006). Stepwise multivariable analyses confirmed TC < 157 mg/dL as an independent prognostic factor for DFS (HR = 1.622; 95% CI = 1.127–2.333; *p* = 0.009), along with stage III–IV disease and P–D.

### 3.5. Nomogram for Overall Survival Prediction

**Figure 4:**
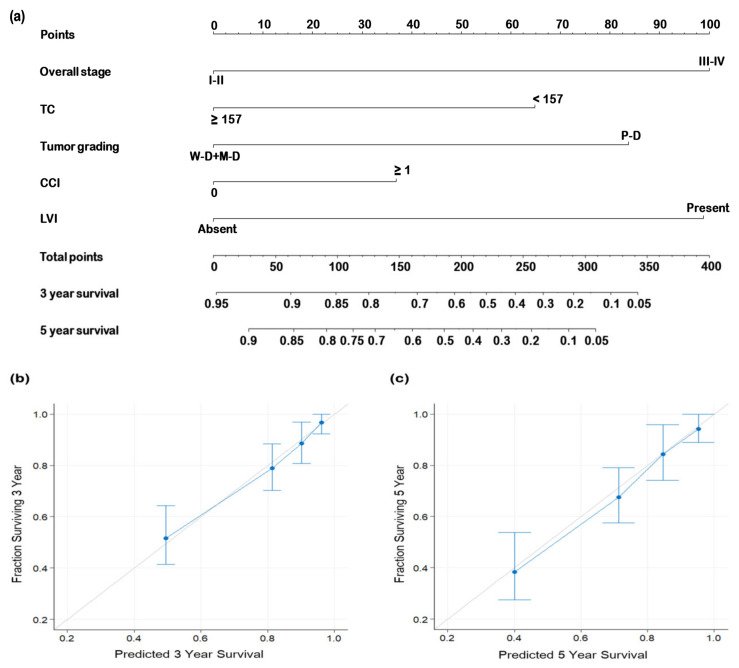
Nomogram for predicting overall survival. (**a**) Multivariable-analysis-derived independent prognostic factors were used to construct a nomogram for predicting overall survival. The contribution of each variable’s risk is illustrated by the length of its line segment and its corresponding points. The total points were derived by summing the points assigned to each individual variable. The line extending from the calculated total points indicates the overall survival probabilities at 3 years and 5 years. Calibration plots were generated for (**b**) 3-year and (**c**) 5-year overall survival rates. The gray 45° line signifies perfectly accurate overall survival predictions, and the blue line represents the actual nomogram-derived predictions. The nomogram’s performance and the 95% confidence intervals for overall survival predictions are illustrated using blue dots and bars, respectively. Abbreviations: LVI, lymphovascular invasion; M–D, moderately differentiated; P–D, poorly differentiated; TC, total cholesterol; W–D, well differentiated.

We constructed a nomogram to predict OS, incorporating independent prognostic factors identified in the multivariable analysis: AJCC stage, TC level, tumor grade, CCI, and LVI (Figure 4). Each variable’s contribution is represented by its corresponding line segment and assigned points. The total points were derived by summing the points for each variable. The line extending from the calculated total points indicates the overall survival probabilities at 3 years and 5 years. We derived a C-index of 0.74 (95% CI: 0.71–0.78) for our nomogram, compared to 0.64 (95% CI: 0.61–0.67) for the TNM staging system, demonstrating the nomogram’s superior discriminative ability in predicting OS. Furthermore, calibration plots showed strong agreement between predicted and observed OS rates at 3 and 5 years (Figure 4b and Figure 4c, respectively). These findings underscore the nomogram’s enhanced predictive performance by incorporating additional clinicopathological factors and its robustness as a tool for individualized survival prediction.

## 4. Discussion

To the best of our knowledge, this study represents the first exploration of the prognostic value of TC in OSCC, and we uncovered several new insights. First, we found that a low preoperative TC level (<157 mg/dL) is significantly associated with older age (≥65 years), advanced tumor stages (T3 or T4), the presence of PNI, and DOI ≥ 10 mm. This suggests that TC may be a useful indicator of tumor aggressiveness in OSCC. Second, the results from multivariable analysis reveal that a low TC level (<157 mg/dL) is an independent predictor of poor OS and DFS. The consistent and significant prognostic value of TC across subgroups highlights its robustness and potential as a reliable biomarker for OSCC, regardless of tumor subsites, tumor or nodal status, resection margins, or DOI. Currently, the TNM staging system is widely used for OSCC prognostication and treatment stratification. However, it does not consider other critical clinicopathological factors of OSCC, such as PNI, LVI, and tumor grade. Compared to the TNM staging system, our constructed nomogram demonstrates superior predictive performance with several practical implications for clinical practice. It incorporates significant prognostic factors beyond the TNM system, facilitates risk stratification to identify high-risk patients who may benefit from intensified adjuvant therapies or closer postoperative surveillance and serves as a valuable tool for shared decision-making by improving communication and aligning treatment strategies with patient-specific risks. These features make the nomogram a valuable complement to traditional TNM staging in clinical practice.

Research shows that low serum TC levels are linked to higher risks of various cancers and higher mortality [12,13]. For example, Ohno et al. assessed preoperative TC levels in 364 patients with renal cell carcinoma who received surgical treatment over a 20-year span; they found that low TC levels were an independent prognostic factor for cancer-specific survival across all stages [23]. In a retrospective study of 1251 patients with gastric cancer, Shin et al. discovered that low TC levels correlated with more postoperative complications, lower OS, and lower recurrence-free survival [13]. Another study on patients with pancreatic cancer found that while serum TC levels decreased shortly after surgery, higher TC levels four weeks post-surgery were associated with better long-term survival [15]. Nevertheless, other previously executed research has reported conflicting results. For example, a retrospective study on early-stage cervical cancer reported that higher TC levels correlated with worse OS rates [11]. A meta-analysis by Li et al. found that high preoperative TC levels were positively correlated with cancer-specific and progression-free survival [24]. Although high TC levels have been reported to accelerate cancer cell growth, serum TC levels tend to decrease as cancer progresses due to the effects of cancer metabolism [12]. This may partly explain the inconsistent results, underscoring the need to consider the timing of TC measurements at cancer diagnosis and the extent of the tumor. Previous research has demonstrated that reprogramming lipid metabolism plays a role in the development and progression of OSCC [25,26]. Our findings further support using preoperative TC as a prognostic marker in OSCC and expanding its clinical applicability in cancer management.

The mechanism by which TC influences the survival of patients with OSCC remains unclear [19]. Rapidly proliferating cancer cells either produce cholesterol in the endoplasmic reticulum or uptake cholesterol from the bloodstream [27]. Studies have shown that patients with OSCC or oral premalignant lesions often have lower blood TC levels compared to healthy individuals [28,29,30], likely due to increased LDL receptor activity that enhances cholesterol uptake for cell membrane production [31]. Additionally, interleukin-6 (IL-6), a pro-inflammatory protein associated with cancer progression, significantly impacts cholesterol metabolism through various pathways [32]. IL-6 alters hepatic cholesterol metabolism by increasing very low-density lipoprotein synthesis, reducing high-density lipoprotein production, and impairing bile acid synthesis by downregulating cholesterol 7α-hydroxylase [33,34]. IL-6 also induces serum amyloid A production, leading to dysfunctional high-density lipoprotein with impaired anti-inflammatory and anti-oxidative properties, and modulates low-density lipoprotein receptor expression [35,36]. Finally, IL-6 alters cholesterol biosynthesis through the JAK/STAT pathway and promotes the uptake of oxidized low-density lipoprotein by macrophages [37,38,39]. The overexpression of IL-6 reduces serum lipid levels and has been linked to advanced tumor grades, larger tumors [40], poor responses to chemoradiotherapy, and shorter survival time in OSCC [41]. Our study aligns with these findings, as low TC (< 157 mg/dL) correlates with greater tumor burden, DOI ≥ 10 mm, and poor OS and DFS. Furthermore, low cholesterol levels can indicate malnutrition, which may affect survival outcomes in OSCC [41]. Severe malnutrition is recognized as an independent risk factor for OS in head and neck cancers [42]. The Controlling Nutritional Status score, a widely used nutritional assessment tool that incorporates serum TC levels, has been reported to predict the prognosis of OSCC [43]. Cholesterol metabolism’s interaction with antitumor immune responses may also contribute to the prognostic significance of TC [44]. Cholesterol enrichment is essential for CD4+ T cell proliferation and activation [45], and it activates nature killer cells against tumors in vivo [46]. Hypocholesterolemia is associated with lower counts of total T cells, CD8+ cells, and circulating lymphocytes, hindering T cell proliferation and inducing autophagy-mediated apoptosis [47,48]. On the other hand, head and neck cancer cells adapt their lipid metabolism to the tumor microenvironment [7], leading to a deficiency in T cell cholesterol and subsequent T cell exhaustion or dysfunction [48]. Overall, low TC may be associated with poor prognoses in OSCC due to factors like increased lipid use by cancer cells, interleukin-6 overexpression, malnutrition, and weakened antitumor immunity. Future studies are needed to explore the precise underlying mechanisms.

Our findings suggest that incorporating preoperative TC measurements into routine assessments may offer valuable clinical benefits. First, as shown in our study, low TC levels are associated with unfavorable survival, suggesting that TC is a valuable marker for risk stratification. Incorporating TC into prognostic tools, such as our proposed nomogram, enables more personalized survival predictions, helping clinicians tailor treatment strategies. Second, statins, which are widely used to lower cholesterol levels, have demonstrated anti-cancer properties, including inducing cancer cell apoptosis, inhibiting tumor growth, and enhancing the effectiveness of both chemotherapy and immunotherapy [49,50,51]. Statin use has been linked to a reduced risk of OSCC among betel nut chewers, indicating its potential anti-cancer effects [52]. In a retrospective study of 602 patients with OSCC, statin use was associated with improved OS and recurrence-free survival, particularly among patients younger than 70 [53]. Therefore, assessing TC levels preoperatively could help identify patients with dysregulated lipid profiles, enabling the potential integration of statin therapy into their treatment regimens. Moreover, high TC levels are a significant risk factor for cardiovascular diseases [54]. Patients with OSCC, particularly those undergoing multimodal therapy, are at elevated risk for cardiovascular complications [55,56]. Preoperative TC assessment can help identify patients at higher cardiovascular risk, enabling early interventions and preventive measures to improve their long-term health outcomes. In addition, low TC levels may indicate malnutrition or metabolic changes linked to systemic inflammation and aggressive tumor biology [57]. Identifying patients with low TC levels could guide nutritional assessments and interventions, such as dietary modifications or supplementation, to enhance metabolic health and improve quality of life. However, further studies are needed to validate these findings and refine their clinical application.

This study has several limitations. First, being a retrospective analysis, this study is inherently subject to biases, including selection and information biases, which may influence the findings. In addition, TC is a dynamic parameter that can fluctuate with cancer progression and treatment. However, we did not include serial TC measurements or comprehensive lipid profiles. Furthermore, although we excluded patients taking statins to minimize potential confounding effects, this exclusion may have introduced selection bias by removing a subset of patients who could differ systematically from those not taking statins. Finally, our study results have not yet been validated with independent datasets. External validation using diverse clinical cohorts and independent datasets is crucial to confirm their generalizability and applicability to broader OSCC populations.

Future prospective studies should include statin-treated patients to evaluate the impact of statin use on TC levels and survival outcomes. Additionally, incorporating serial TC measurements may allow for assessing dynamic TC changes and their correlation with tumor progression, treatment response, and survival outcomes. Larger, multicenter cohorts with diverse populations and comprehensive lipid profiles, including low-density lipoprotein, high-density lipoprotein, and triglycerides, may enhance the understanding of lipid metabolism’s role in OSCC prognosis. Integrating biomarker analysis, such as inflammatory markers like IL-6, may uncover the mechanisms linking TC and cancer outcomes. Additionally, randomized controlled trials assessing cholesterol-modulating interventions, such as statins or dietary modifications, may clarify the clinical relevance of TC as a prognostic marker and guide therapeutic strategies. By addressing the above-mentioned limitations, future studies may provide stronger evidence for the prognostic role of TC in OSCC, clarify its biological underpinnings, and help establish standardized protocols for its integration into clinical practice.

## 5. Conclusions

Our study demonstrates that low preoperative TC levels (<157 mg/dL) are independently associated with poorer OS and DFS in patients with OSCC. The nomogram we developed, incorporating TC and key clinicopathological factors, showed superior predictive accuracy compared to the TNM staging system (C-index: 0.74 vs. 0.64). These findings highlight the potential of TC as a prognostic biomarker and emphasize the need for routine preoperative cholesterol assessment in patients with OSCC. Future research, including prospective studies and randomized controlled trials, is essential to validate the role of TC in OSCC prognosis and to explore the integration of cholesterol-modulating strategies, such as statin therapy, into personalized treatment approaches.

## Figures and Tables

**Table 1:** Baseline characteristics and correlations between clinicopathological variables and total cholesterol.

Variables	Total, *N* (%)	Total Cholesterol ≥ 157 (*n* = 230)	Total Cholesterol < 157 (*n* = 80)	*p*
Age (years)				0.010 ^a^
<65	196 (63.2%)	155 (67.4%)	41 (51.3%)	
≥65	114 (36.8%)	75 (32.6%)	39 (48.8%)	
Sex				0.581 ^a^
Men	280 (90.3%)	209 (90.9%)	71 (88.8%)	
Women	30 (9.7%)	21 (9.1%)	9 (11.3%)	
Tumor location				0.282 ^a^
Tongue	120 (38.7%)	95 (41.3%)	25 (31.3%)	
Buccal mucosa	101 (32.6%)	72 (31.3%)	29 (36.3%)	
Others	89 (28.7%)	63 (27.4%)	26 (32.5%)	
Personal Habits *				0.767 ^a^
No exposure	38 (12.3%)	27 (11.7%)	11 (13.8%)	
One exposure	16 (5.2%)	11 (4.8%)	5 (6.3%)	
Two or more exposures	256 (82.6%)	192 (83.5%)	64 (80.0%)	
AJCC stage				0.110 ^a^
I	66 (21.3%)	53 (23.0%)	13 (16.3%)	
II	43 (13.9%)	36 (15.7%)	7 (8.88%)	
III	45 (14.5%)	29 (12.6%)	16 (20.0%)	
IV	156 (50.3%)	112 (48.7%)	44 (55.0%)	
T status				0.020 ^a^
T1−T2	143 (46.1%)	115 (50.0%)	28 (35.0%)	
T3−T4	167 (53.9%)	115 (50.0%)	52 (65.0%)	
N status				0.110 ^a^
N0	201 (64.8%)	155 (67.4%)	46 (57.5%)	
N+	109 (35.2%)	75 (32.6%)	34 (42.5%)	
Presence of PNI				0.049 ^a^
No	231 (74.5%)	178 (77.4%)	53 (66.3%)	
Yes	79 (25.5%)	52 (22.6%)	27 (33.8%)	
Presence of ENE				0.330 ^a^
No	248 (80.0%)	187 (81.3%)	61 (76.3%)	
Yes	62 (20.0%)	43 (18.7%)	19 (23.8%)	
Presence of LVI				0.183 ^a^
No	289 (93.2%)	217 (94.3%)	72 (90.0%)	
Yes	21 (6.8%)	13 (5.7%)	8 (10.0%)	
Tumor grading				0.296 ^a^
W−D/M−D	277 (89.4%)	208 (90.4%)	69 (86.3%)	
P−D	33 (10.6%)	22 (9.6%)	11 (13.8%)	
Closest resection margin				0.548 ^a^
≥5 mm	225 (72.6%)	169 (73.5%)	56 (70.0%)	
<5 mm	85 (27.4%)	61 (26.5%)	24 (30.0%)	
DOI ≥ 10 mm				0.018 ^a^
No	167 (53.9%)	133 (57.8%)	34 (42.5%)	
Yes	143 (46.1%)	97 (42.2%)	46 (57.5%)	
Treatment modality				0.124 ^a^
Surgery only	149 (48.1%)	118 (51.3%)	31 (38.8%)	
Surgery then RT	44 (14.2%)	29 (12.6%)	15 (18.8%)	
Surgery then CRT	117 (37.7%)	83 (36.1%)	34 (42.5%)	
CCI				0.112 ^a^
0	167 (53.9%)	130 (56.5%)	37 (46.3%)	
≥1	143 (46.1%)	100 (43.5%)	43 (53.8%)	
WBC (×10^3^/μL), median (IQR)	7.80 (6.28−9.70)	7.70 (6.20−9.50)	8.10 (6.33−10.30)	0.417 ^b^
Cholesterol (mg/dL), median (IQR)	179 (156−208)	189 (176−221)	140 (126−148)	<0.001 ^b^

AJCC, American Joint Committee on Cancer; CCI, Charlson Comorbidity Index; CRT, chemoradiotherapy; DOI, depth of invasion; ENE, extranodal extension; IQR, interquartile range; LVI, lymphovascular invasion; M−D, moderately differentiated; P−D, poorly differentiated; PNI, perineural invasion; RT, radiotherapy; W−D, well differentiated; WBC, white blood cell. ^a^ Chi-square test. ^b^ Mann–Whitney U test. * Personal habits include cigarette smoking, alcohol consumption, and betel nut chewing.

**Table 2:** Univariable and multivariable analyses for overall survival.

Variables		Univariable Analysis		Multivariable Analysis	
		HR (95% CI)	*p*	HR (95% CI)	*p*
Sex	Men vs. Women	1.645 (0.715–3.783)	0.241		
Age (years)	≥65 vs. <65	0.763 (0.483–1.204)	0.245		
AJCC stage	III–IV vs. I–II	3.713 (2.056–6.708)	<0.001	2.405 (1.165–4.967)	0.018
Presence of PNI	Yes vs. no	2.450 (1.585–3.789)	<0.001		
Presence of LVI	Yes vs. no	3.886 (2.091–7.221)	<0.001	2.892 (1.507–5.551)	0.001
Tumor grading	P–D vs. W–D/M–D	2.851 (1.669–4.870)	<0.001	2.111 (1.189–3.746)	0.011
Closest margin (mm)	<5 vs. ≥5	1.605 (1.031–2.498)	0.036		
Adjuvant therapy	Yes vs. no	3.090 (1.922–4.969)	<0.001		
CCI	≥1 vs. 0	1.570 (1.023–2.410)	0.039	1.623 (1.051–2.506)	0.029
Total cholesterol	<157 vs. ≥157	2.463 (1.601–3.792)	<0.001	2.114 (1.368–3.269)	0.001

AJCC, American Joint Committee on Cancer; CCI, Charlson Comorbidity Index; CI, confidence interval; HR, hazard ratio; LVI, lymphovascular invasion; M–D, moderately differentiated; P–D, poorly differentiated; PNI, perineural invasion; W–D, well differentiated. Variance inflating factor: AJCC stage: 1.006; LVI: 1.013; tumor grading: 1.005; CCI: 1.014; total cholesterol: 1.002.

**Table 3:** Univariable and multivariable analyses for disease-free survival.

Variables		Univariable Analysis		Multivariable Analysis	
		HR (95% CI)	*p*	HR (95% CI)	*p*
Sex	Men vs. Women	1.509 (0.810–2.812)	0.195		
Age (years)	≥65 vs. <65	0.696 (0.485–1.018)	0.053		
AJCC stage	III–IV vs. I–II	2.126 (1.442–3.134)	<0.001	2.242 (1.391–3.615)	0.001
Presence of PNI	Yes vs. no	1.359 (0.938–1.970)	0.105		
Presence of LVI	Yes vs. no	1.966 (1.083–3.568)	0.026		
Tumor grading	P–D vs. W–D/M–D	1.785 (1.109–2.873)	0.017	1.732 (1.053–2.848)	0.030
Closest margin (mm)	<5 vs. ≥5	1.384 (0.971–1.972)	0.072		
Adjuvant therapy	Yes vs. no	1.526 (1.088–2.141)	0.014		
CCI	≥1 vs. 0	0.986 (0.704–1.380)	0.934		
Total cholesterol	<157 vs. ≥157	1.630 (1.144–2.324)	0.006	1.622 (1.127–2.333)	0.009

AJCC, American Joint Committee on Cancer; CCI, Charlson Comorbidity Index; CI, confidence interval; HR, Hazard ratio; LVI, lymphovascular invasion; M–D, moderately differentiated; P–D, poorly differentiated; PNI, perineural invasion; W–D, well differentiated. Variance inflating factor: AJCC stage: 1.023; tumor grading: 1.009; total cholesterol: 1.052.

## Data Availability

Data from the current study are available from the corresponding author upon reasonable request.
